# Subcellular analysis of nuclear and cytoplasmic redox indices differentiates breast cancer cell subtypes better than nuclear-to-cytoplasmic area ratio

**DOI:** 10.1117/1.JBO.27.8.086001

**Published:** 2022-08-09

**Authors:** Annemarie Jacob, He N. Xu, Andrea L. Stout, Lin Z. Li

**Affiliations:** a University of Pennsylvania, Perelman School of Medicine, Department of Radiology, Britton Chance Laboratory of Redox Imaging, Philadelphia, Pennsylvania, United States; b University of Pennsylvania, Perelman School of Medicine, Institute of Translational Medicine and Therapeutics, Philadelphia, Pennsylvania, United States; c University of Pennsylvania, Perelman School of Medicine, Department of Cell and Developmental Biology, Philadelphia, Pennsylvania, United States

**Keywords:** optical redox imaging, aggressiveness, triple-negative breast cancer, mitosis, reduced nicotinamide adenine dinucleotide, flavin adenine dinucleotide

## Abstract

**Significance:**

Stratification of malignancy is valuable for cancer treatment. Both optical redox imaging (ORI) indices and nuclear-to-cytoplasmic volume/area ratio (N:C ratio) have been investigated to differentiate between cancers with varying aggressiveness, but these two methods have not been directly compared. The redox status in the cell nucleus has not been studied by ORI, and it remains unknown whether nuclear ORI indices add new biological information.

**Aim:**

We sought to compare the capacity of whole-cell and subcellular ORI indices and N:C ratio to differentiate between breast cancer subtypes with varying aggressiveness and between mitotic and nonmitotic cells.

**Approach:**

ORI indices for whole cell, cytoplasm, and nucleus as well as the N:C area ratio were generated for two triple-negative (more aggressive) and two receptor-positive (less aggressive) breast cancer cell lines by fluorescence microscopy.

**Results:**

We found positive correlations between nuclear and cytoplasmic ORI indices within individual cells. On average, a nuclear redox status was found to be more oxidized than cytoplasm in triple-negative cells but not in receptor-positive cells. Whole-cell and subcellular ORI indices distinguished between the receptor statuses better than the N:C ratio. However, N:C ratio was a better differentiator between nonmitotic and mitotic triple-negative cells.

**Conclusions:**

Subcellular ORI analysis differentiates breast cancer subtypes with varying aggressiveness better than N:C area ratio.

## 1 Introduction

Breast cancer exhibits diverse prognoses across and within subtypes. There is an unmet need for biomarkers that can detect malignant or aggressive tumors to aid in the selection of treatment strategies. Optical metabolic metrics have been under development in (pre)cancer imaging based on the endogenous fluorescence of oxidized flavoproteins (Fp containing flavin adenine dinucleotide, i.e., FAD) and reduced nicotinamide adenine dinucleotide (NADH).^1^^–^^6^ The optical redox ratio (ORR, defined as Fp/(Fp + NADH) in this study), an indicator of the mitochondrial metabolic and redox status, has been investigated as a potential biomarker for cancer progression and treatment response.^6^^–^^12^

In particular, ORR has been utilized to predict the invasive potential or aggressiveness of breast cancer cells *in vitro*. It was found that increased ORR correlated with higher invasive potential^13^^,^^14^ and the knockdown of an oncogene decreased the redox ratio with a concomitant decrease of invasive potential,^15^ indicating that ORR is an indicator of breast cancer aggressiveness *in vitro*. Although most of the Fp and NADH signals come from the mitochondria, these previous analyses were performed with intensity thresholding and global averaging of the whole cell without partitioning into subcellular compartments. However, it is known that the nucleus also contains FAD and NADH and emits low levels of Fp and NADH signals.^16^^–^^18^ The measurement of the optical redox indices (Fp, NADH, and ORR) from the subcellular compartments, such as cytoplasm and nucleus, may provide additional information that could be linked to breast cancer cell aggressiveness.

Therefore, a major goal of this study is to examine the optical redox imaging (ORI) index differences between the whole cell, cytoplasm, and nucleus and to determine whether the compartmental ORI indices can differentiate between breast cancer cell subtypes exhibiting varying levels of aggressiveness.

We also aimed to compare the utility of ORI indices and nuclear-to-cytoplasmic area (N:C) ratio to differentiate between breast cancer cell types. The N:C ratio based on the ratio of cross-sectional areas has been investigated as an indicator of malignancy in several types of cancers.^19^^–^^21^ A higher N:C ratio is regarded as an indicator of an increased amount of chromatin in cancerous cells.^22^ However, a study of human cheek lesions indicated that the N:C ratio in basal or spinous cells has no value as a predictor of the malignant potential of cheek lesions.^20^ Thus, the examination of morphological changes alone may not suffice as a biomarker of malignant potential. In addition, cancer cells are constantly proliferating under normal cell culture conditions, and they are in different stages of cell cycles at any given time point. We sought to investigate whether the ORI indices and the N:C ratio can differentiate between dividing (mitotic) and nondividing (nonmitotic) breast cancer cells.

## 2 Materials and Methods

We examined four breast cancer cell lines, i.e., HCC1806 (triple-negative breast cancer A), MDA-MB-231 (triple-negative breast cancer B), MCF7 (luminal type A), and BT474 (luminal type B). Their classifications were determined by previous studies.^23^ Triple-negative breast cancers (TNBCs) consist of negative phenotypes of the estrogen receptor (ER), progesterone receptor (PR), and human epithelial receptor 2 (HER2). BT474 has positive phenotypes for all of these receptors, and MCF7 is an ${\mathrm{ER}}^{+}/{\mathrm{PR}}^{+}/{\mathrm{HER}2}^{-}\mathrm{cell}$ line. For the purpose of this study, the two TNBC cell lines were defined as the triple-negative group and the luminal cell lines as the receptor-positive group. In general, TNBC patients are more resistant to chemotherapy, have the poorest prognosis among breast cancer subtypes, and exhibit higher aggressiveness or invasive/metastatic potential than receptor-positive breast cancer.^23^

Breast cancer cells were cultured in RPMI 1640 Medium (Gibco, Thermo Fisher Scientific, Waltham, Massachusetts, United States) supplemented with 10% fetal bovine serum and maintained at 37°C with 5% ${\mathrm{CO}}_{2}$. The cells were passaged at ∼80% confluency. The cells were seeded in 35-mm glass-bottom dishes (with 20-mm bottom well; Product #: D35-20-1.5-N, Cellvis, Sunnyvale, California, United States) at a density of 70,000 cells/500  μL/dish and incubated for ∼24  h and reached a confluency of about 50%. Roughly 45 min prior to imaging, cells were rinsed twice with phosphate-buffered saline (PBS) with calcium and magnesium (Thermo Fisher Scientific, Waltham, Massachusetts, United States), incubated in 1 mL live cell imaging solution (Thermo Fisher Scientific, Waltham, Massachusetts, United States), and supplemented with 11 mM of glucose and 2.0 mM of L-glutamine (final concentrations).

To take live cell optical redox images, cell dishes were maintained in an environmental chamber surrounding the entire microscope for temperature control set at 37°C and imaged with the Zeiss Axio Observer 7 wide-field microscope (Carl Zeiss Microscopy LLC, White Plains, New York, United States), available in the Cell and Developmental Biology Microscopy Core Facility at the University of Pennsylvania. The scope is equipped with a Colibri 7 light-emitting diode (LED) light source and a Zeiss Axiocam 702 monochrome complementary metal oxide semiconductor (CMOS) camera. All imaging was conducted with an objective of 20× (Numerical Aperture 0.8), image matrix 1920×1216, pixel size $0.293\times 0.293\;{\mu m}^{2}$, bit depth 14 bit, and gain 1. The DAPI (4',6-diamidino-2-phenylindole) channel, used for the NADH measurement in this study, is illuminated by the 385-nm LED at an intensity level of 25%, with a bandpass excitation (Ex) filter of 370 to 400 nm and emission (Em) filter of 414 to 450 nm, and an exposure time of 2 s. The enhanced green fluorescent protein (EGFP) channel, designated for the Fp measurement, is illuminated by the 475-nm LED at an intensity level of 40%, with a bandpass Ex filter of 450 to 488 nm and an Em filter of 500 to 530 nm, and an exposure time of 3 s. All imaging was accomplished during a period of ∼3 months.

To demonstrate that the microscope remains stable with no significant system drift during this period, the illumination power of four LED light sources (385, 475, 555, and 630 nm) was measured by a power meter and found to be very stable with the coefficient of variations (CVs) within 1% for all wavelengths and levels of intensities [Figs. S1(a) and S1(b) and Table S1 in the Supplemental Material]. The temporal power drifts within 100 days were < 2% for the light sources used in this study. Furthermore, 100  μM NADH and FAD aqueous solutions and blank PBS solutions were imaged repeatedly for 3 months using the same experimental protocol and instrument settings (Supplemental Material). The fluorescence signals of all solution samples were found to be stable without significant time-dependent drifts [Figs. S1(c) and S1(d) and Table S2 in the Supplemental Material]. The PBS signals had CVs within 2% reflecting the excellent stability of the microscope system. The nonsignificant trends of signal decay of NADH and FAD solutions, 7% and 4%, respectively, within 100 days probably indicate a slight chemical decay during the long-term storage in a −80°C freezer (Supplemental Material). The fluorescence CVs of both NADH and FAD solutions were found to be within 4%. In comparison, the biological sample variations are usually up to 10% to 20% or more. In short, our Zeiss microscope system is very stable for the period of 3 months, and its calibration with standard references is not necessary for the purpose of this study.

For each cell line, three dishes were imaged, and three fields of view (FOVs) were selected per dish at random for imaging and further analysis without binning. The raw fluorescence intensities from the microscope were reported in arbitrary units. A custom MATLAB^®^ (Version 2018b, The MathWorks, Inc., Natick, Massachusetts, United States) program was developed to analyze ORI images and to extract the key data, such as the intensities of NADH and Fp and the ORR Fp/(Fp+NADH) for individual cells without apparent nuclear division. Fifteen cells with clearly visible nuclear boundaries based on NADH raw images were randomly selected across different FOVs from each of the three dishes. These cells were then individually analyzed by manually drawing a region of interest (ROI) on raw NADH monochrome images, as shown in Fig. S2 in the Supplemental Material, with both the whole cell and nucleus drawn. Cytoplasm ROI was obtained by subtracting the nuclear region from the whole-cell region. For each cell selected for analysis, we generated NADH and Fp images after subtraction of the background and thresholding individual pixels at a signal intensity-to-background noise ratio (SNR) of 7.5, which was satisfied for both channels. The redox ratio image was obtained from Fp and NADH images on a pixel-by-pixel basis. We then determined the average redox indices in the three ROIs: the whole cell, the nucleus, and the cytoplasm. These indices were, respectively, averaged for each FOV using means of NADH, Fp, and the redox ratio of individual cells and then averaged across FOV (N=3) and dishes (N=3) to find the overall group means and standard deviations (SDs) for each cell line.

Mitotic cells were chosen randomly and visually based on nuclear morphology displaying apparent division in the later stages of cell mitosis, such as telophase [Figs. S2(d)–S2(f) in the Supplemental Material]. Fifteen mitotic cells were analyzed per dish for both MDA-MB-231 and HCC1806. The same analysis was not performed for BT474 and MCF7 as they have doubling times of ∼2 days or more in RPMI 1640 medium^24^ and few cells were observed to have nuclear division during imaging. The N:C ratio was determined for individual cells. We calculated the area of the whole cell and the nucleus based on the number of nonzero pixels after signal thresholding using the MATLAB method *bwarea*. The cytoplasmic area was calculated based on the subtraction of the nuclear area from the area of the entire cell.

We performed ORI and N:C ratio analyses using a dish-based analysis or cell-based analysis. Dish-based analysis treated each dish as an independent replicate (N=3). Cell-based analysis treated each cell as an independent replicate (N=45). Comparison between the triple-negative group and the receptor-positive group was achieved by pooling the data from both HCC1806 and MDA-MB-231 cell lines (N=6 for dish-based, N=90 for cell-based analysis) and separately pooling the data from receptor-positive cell lines (BT474 and MCF7).

### 2.1 Statistical Analysis

Significance between three or more groups was calculated using Prism 9 (San Diego, California, United States) to run one-way Brown–Forsythe analysis of variance tests followed by Dunnett’s T3 tests to correct for multiple comparisons with p<0.05 considered as significant. Welch’s t-test was performed for comparison between two groups with unequal variances. Significant differences are depicted as: p****<0.0001, p***<0.001, p**<0.01, and p*<0.05. Considering the slight system variation for the Zeiss microscope aforementioned, only significant changes of NADH or Fp signals of more than 15% were considered reliable and reported. Linear regressions were performed by Prism 9. The significance of slope differences was determined by an analysis of covariance using a t-test assuming unequal variances. Data were presented as mean ± SD.

## 3 Results

### 3.1 Comparison of the Redox Indices Between Cellular Compartments

**Fig. 1:**
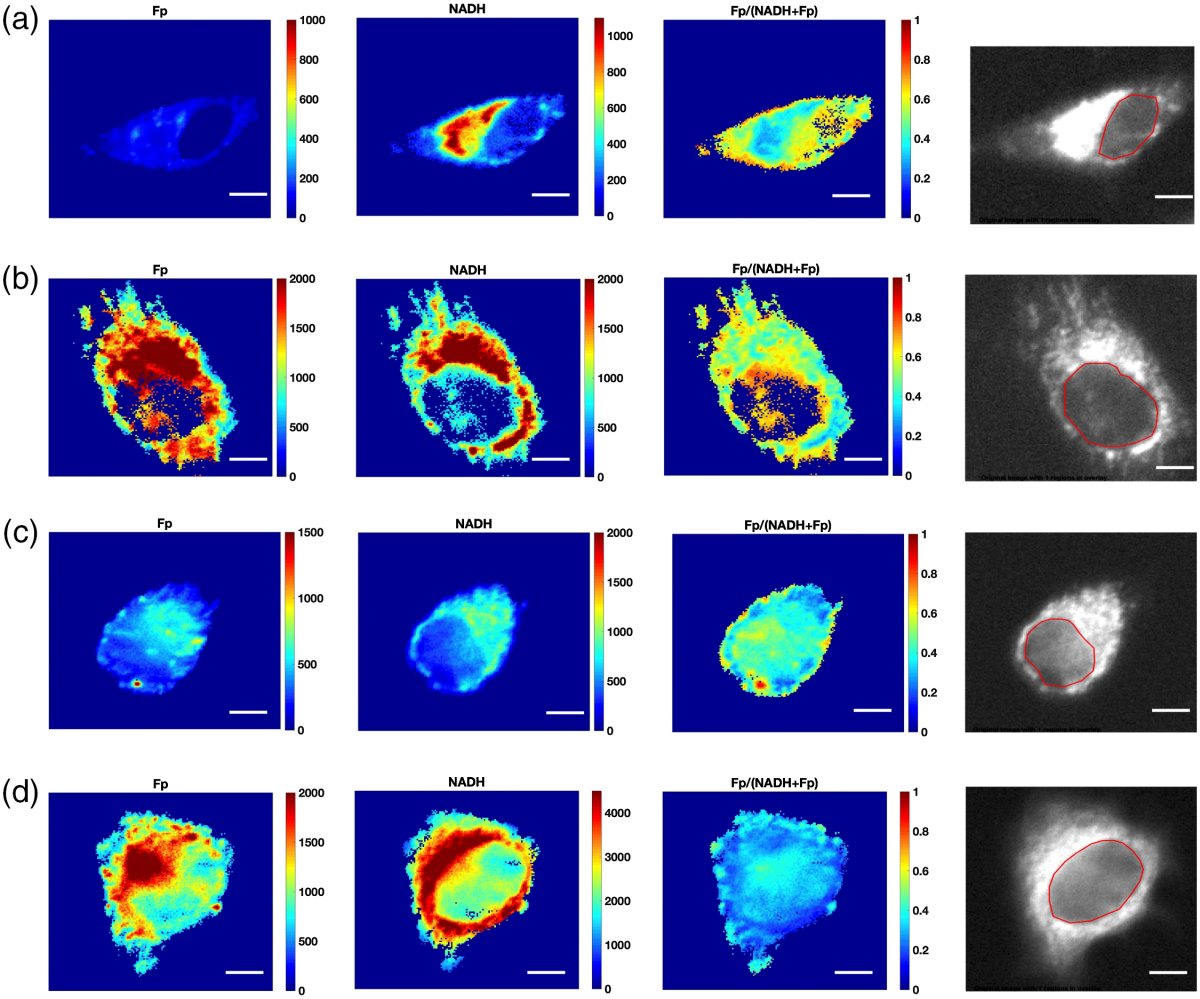
Typical pseudocolor images of Fp, NADH, redox ratio Fp/(NADH + Fp), and black-white NADH raw images from individual breast cancer cells: (a) MDA-MB-231, (b) HCC1806, (c) BT474, and (d) MCF7. The red circles in the black-white images identify the nuclear regions. Scale bars=10  μm.

**Fig. 2:**
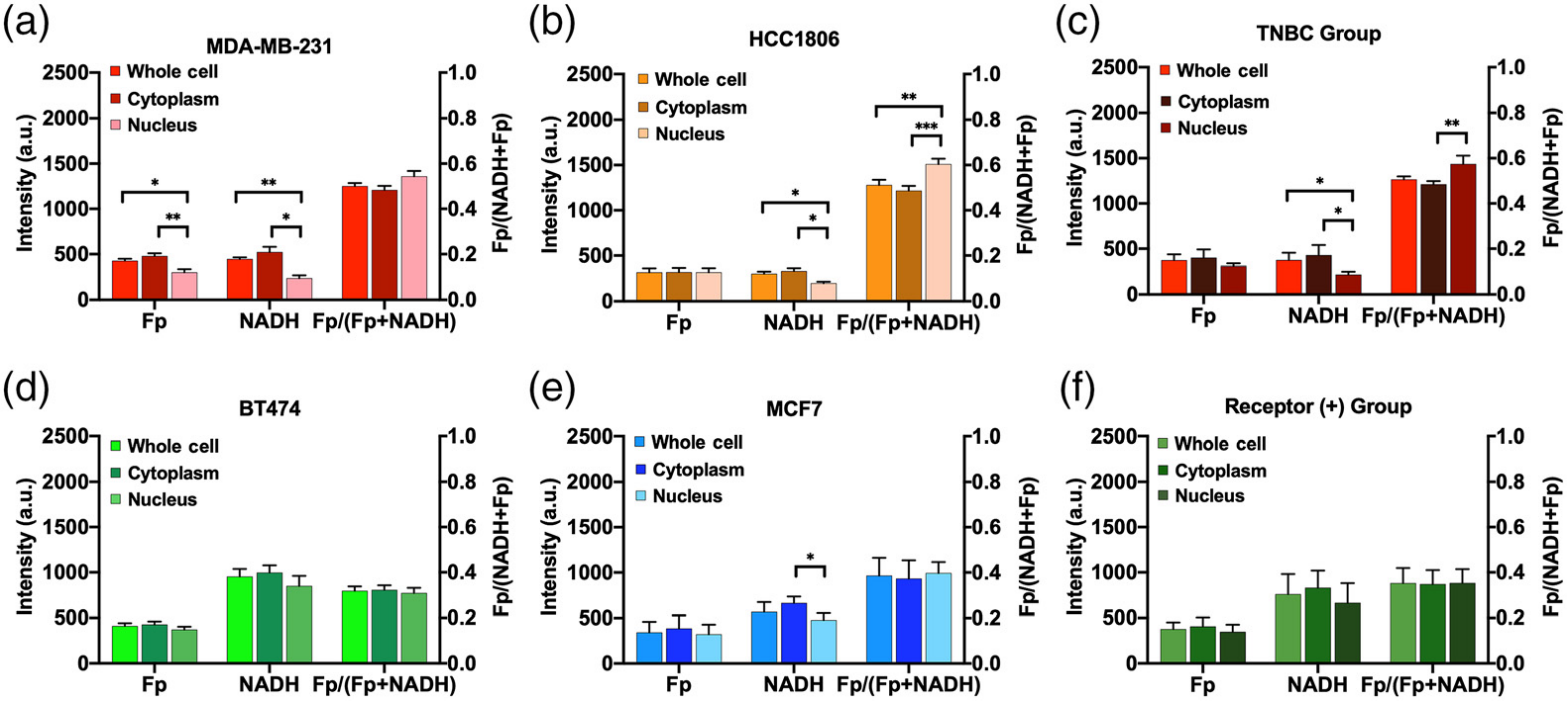
Comparison of the whole cell, cytoplasm, and nucleus with respect to redox indices using a dish-based analysis (N=3, mean ± SD): (a) MDA-MB-231, (b) HCC1806, (c) TNBC group, (d) BT474, (e) MCF7, and (f) receptor-positive group. p***<0.001, p**<0.01, and p*<0.05.

Figure 1 shows the typical optical redox images of individual cells from each breast cancer cell line. The nuclear regions were identified on the black-white images of NADH raw data. The mean SNRs of NADH and Fp signals are >9 in the nucleus and ≥13 in the cytoplasm of each cell line (Table S3 in the Supplemental Material). Figure 2 compared the redox indices of Fp, NADH, and redox ratio Fp/(Fp + NADH) between cell compartments within individual cell lines and within receptor status groups. Both TNBC cell lines exhibited a significant decrease of NADH in the nucleus compared to the whole cell or cytoplasm [Figs. 2(a) and 2(b)]. The redox ratio was the highest in the nucleus among all three compartments. These observations were reconfirmed by the pooled analysis of the triple-negative group [Fig. 2(c)] showing ∼49% decrease in NADH and ∼18% increase in the redox ratio in the nucleus compared to the cytoplasm.

For the receptor-positive group, BT474 and MCF7 lines experienced no significant differences between compartments for all redox indices, except that the nuclear compartment of MCF7 had a significant decrease of 28% in NADH compared to the cytoplasm [Figs. 2(d) and 2(e)]. The pooling of receptor-positive lines showed no significant differences observed between compartments for all indices [Fig. 2(f)].

**Fig. 3:**
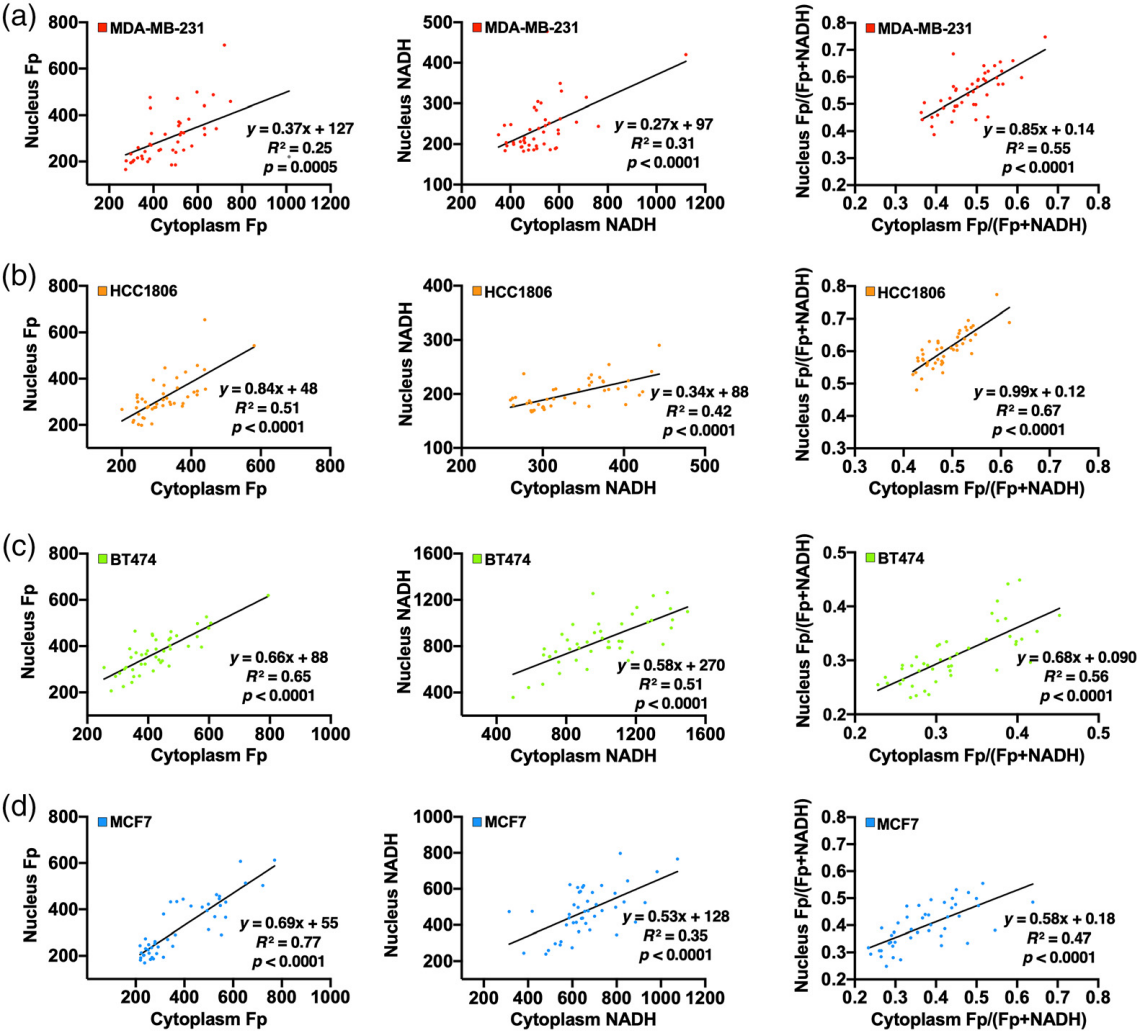
Fp, NADH, and Fp/(Fp + NADH) correlation plots between cytoplasm and nucleus for individual cells of (a) MDA-MB-231, (b) HCC1806, (c) BT474, and (d) MCF7.

**Fig. 4:**
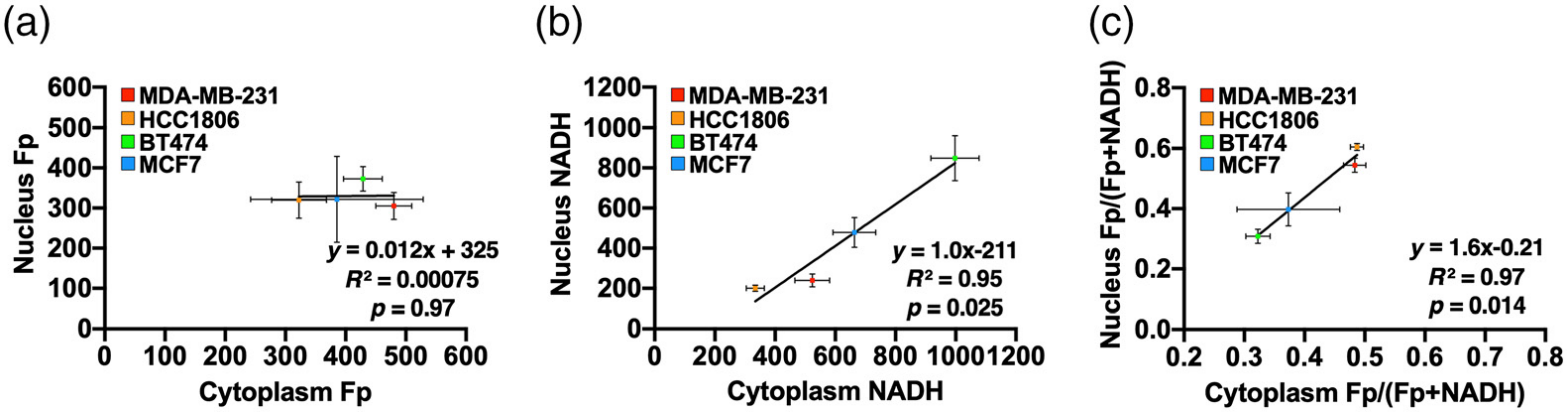
Correlation plots of redox indices between cytoplasm and nucleus across the four cell lines: (a) Fp, (b) NADH, and (c) Fp/(Fp + NADH). The average indices of each cell line were obtained with dish-based analysis. Mean ± SD.

There were also common features observed across all cell lines. Fp, NADH, and the redox ratio exhibited no significant differences between the whole cell and cytoplasm. For each of the four cell lines, we found significant correlations between nuclear and cytoplasmic compartments for the three redox indices of individual cells (Fig. 3); consistent with that, the redox statuses are not independent or isolated between nucleus and cytosol. By pooling the cell-line averages together, we identified significant positive correlations between nucleus and cytosol for NADH intensity and the redox ratio across the four cell lines ($R^{2}\geq 0.95$,p<0.05, Fig. 4). Consistently, the NADH and redox ratio of both subcellular compartments correlate positively and significantly with the corresponding whole-cell indices on the cell-line basis (Fig. S3 in the Supplemental Material). These results suggested that NADH redox status (i.e., NADH and the redox ratio) exhibits a connection between nuclear and cytosolic compartments, which is common across the four breast cancer cell lines. In contrast, for Fp across the four cell lines, there were no significant correlations between nuclear Fp and cytoplasmic Fp and between nuclear Fp and whole-cell Fp, and the positive correlation between cytoplasmic Fp and whole-cell Fp was also marginal (p=0.05; Fig. 4 and Fig. S3 in the Supplemental Material). Thus, nuclear Fp may correlate with cytosolic Fp within individual cells of each cell line (Fig. 3), but no such correlation was found across the four cell lines (Fig. 4).

### 3.2 Whole-Cell and Subcellular Redox Differences Between Cell Lines or Between Receptor Statuses

We examined the differences in the redox indices between cell lines within each compartment using the dish-based analysis (Fig. S4 in the Supplemental Material). Fp signals exhibited no significant difference among the four cell lines in the whole cell and nucleus except a lower cytoplasmic Fp was found in HCC1806 than in MDA-MB-231 cells. However, there were more significant differences in NADH among the four cell lines, with BT474 having the highest value in all compartments. Furthermore, the receptor-positive BT474 and MCF7 lines had or tended to have higher NADH intensity than TNBC lines. Across all compartments, significantly higher redox ratios were observed for HCC1806 and MDA-MB-231 compared to BT474, with an increasing trend compared to MCF7. Figure S5 in the Supplemental Material demonstrates that a cell-based analysis leads to more significant differences between cell lines across all redox indices in all three cell regions due to increased sample size (N=45), with the general patterns of change similar to the dish-based analysis. Highly significant linear correlations, with slopes ∼1 and $R^{2}>0.93$, were found between the dish- and cell-based redox indices (Fig. S6 in the Supplemental Material) demonstrating the consistency between the two analyses.

**Fig. 5:**
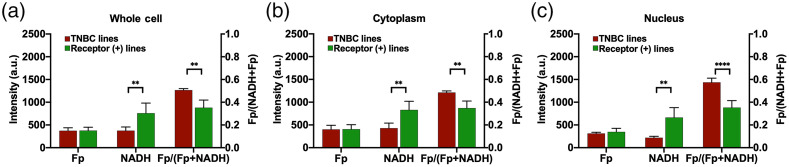
Comparison between TNBC and receptor-positive (+) cell lines for redox indices using dish-based analysis (N=6, mean ± SD): (a) whole cell, (b) cytoplasm, and (c) nucleus. p****<0.0001 and p**<0.01.

We further compared the redox differences between two groups of different receptor statuses (Fig. 5). No difference was observed between Fp levels in all compartments. Significantly lower NADH intensity and higher redox ratios were demonstrated for the whole cell, cytoplasm, and nucleus in the triple-negative than in the receptor-positive group. The whole-cell or cytoplasmic NADH was halved whereas the redox ratio increased by ∼40% in the triple-negative group compared to the receptor-positive group. In contrast, the nuclear NADH was reduced by ∼70%, and the redox ratio was 63% higher in the triple-negative group compared to the receptor-positive group. This marks the ability of both whole-cell and subcellular optical redox indices to differentiate between receptor statuses associated with breast cancer aggressiveness, and the NADH and redox ratio in the nucleus appear to differ more between the receptor statuses than the corresponding indices of cytoplasm or whole cell.

### 3.3 Differences in the N:C Ratios Between Cell Types or Lines

**Fig. 6:**
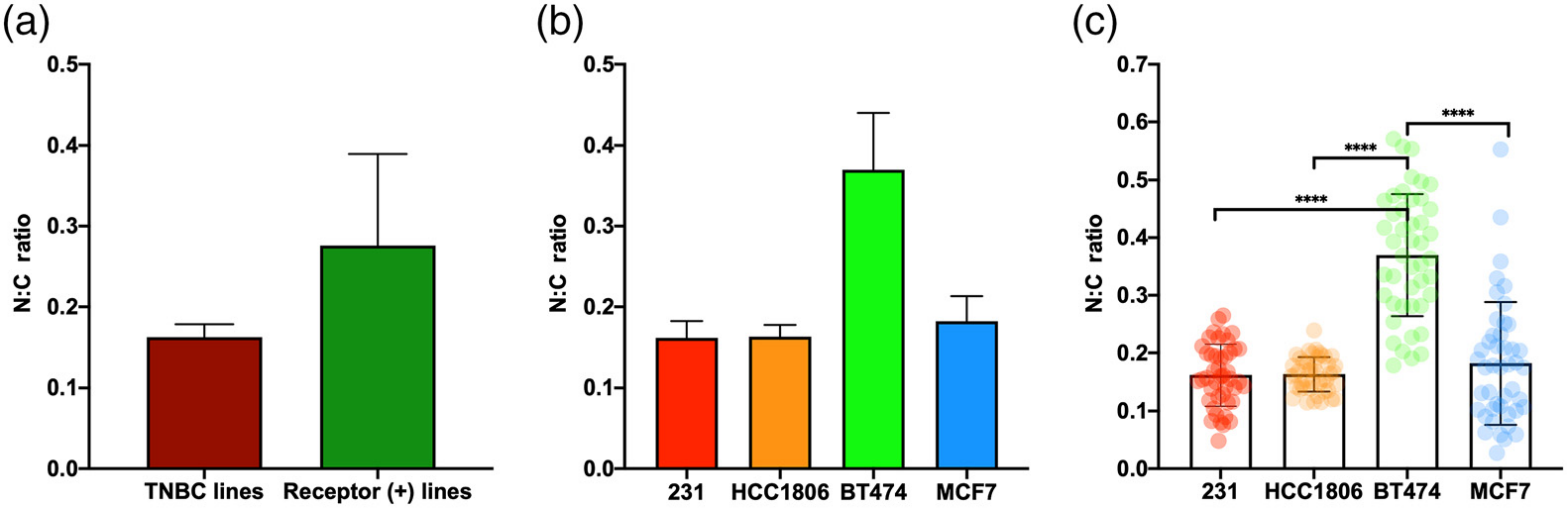
N:C ratios in breast cancer cells: (a) comparison between TNBC and receptor-positive (+) cell lines using pooled dish-based analysis (N=6), (b) dish-based comparison (N=3) between all four cell lines, and (c) cell-based comparison (N=45) between all four cell lines. p****<0.0001. Mean ± SD.

Using the dish-based analysis, a comparison of receptor-positive cell lines to TNBC cell lines marked an increasing trend in the N:C ratio [Fig. 6(a)], and this increase may be attributed to BT474 because when examining the individual cell lines, an increasing trend of N:C ratio of BT474 was observed, whereas MCF7 had similar N:C ratios to MDA-MB-231 and HCC1806 [Fig. 6(b)]. However, there were no significant differences in N:C ratio between all cell lines. When using the cell-based analysis, there were significant increases in N:C ratio for BT474 with respect to MDA-MB-231, HCC1806, and MCF7, but there were no differences between MCF7 and the triple-negative cell lines [Fig. 6(c)]. By pooling cell-based data according to the receptor status, the triple-negative group has a significantly lower N:C ratio than the receptor-positive group (0.16±0.04 versus 0.28±0.14, p<0.0001, n=90). However, Cohen’s effect size of N:C ratio is smaller than the effect size of ORI indices shown in Fig. 5. Taken together, the N:C ratio appeared not to correlate with the breast cancer receptor status or aggressiveness using the dish-based analysis. Although the pooled cell-based analysis detected a significant correlation of N:C ratio with receptor status, this correlation appeared to be weaker than that of ORI indices with receptor status.

### 3.4 Differences in the Redox Indices and N:C Ratios Between Mitotic and Nonmitotic Cells

**Fig. 7:**
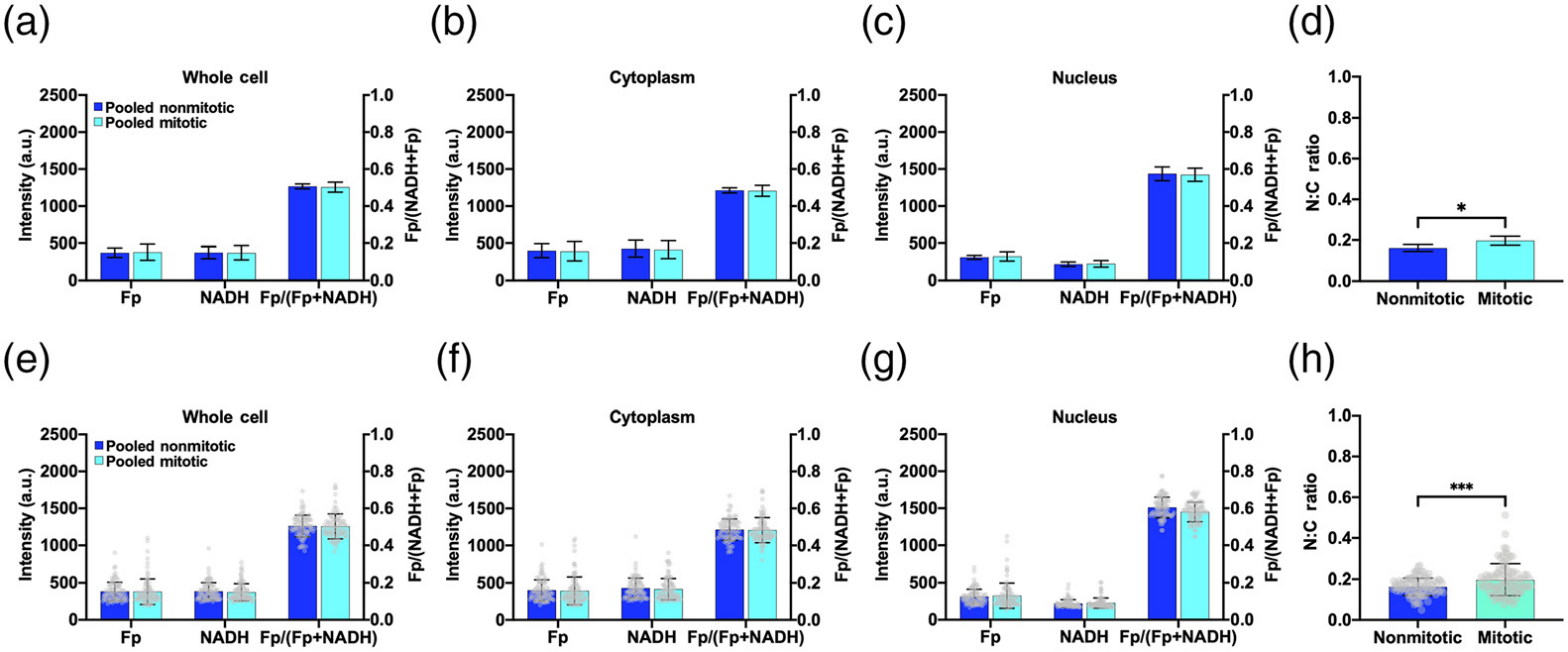
Comparison of nonmitotic and mitotic cells by pooling HCC1806 and MDA-MB-231 cells. (a) Dish-based analysis (N=6) of the whole cell, cytoplasm, and nucleus redox indices and N:C ratio. (b) Cell-based analysis (N=90) of the whole cell, cytoplasm, and nucleus redox indices and N:C ratio. The lower nuclear redox ratio in mitotic cells compared to nonmitotic cells was statistically significant (p<0.001), but the differences did not exceed the reliability threshold of 15% set in this study. p*<0.05 and p***<0.001. Mean ± SD.

Using visible nuclear division as the criterion for mitosis, we investigated the redox and N:C ratio differences between nonmitotic and mitotic cells for the two TNBC cell lines. For both redox indices and N:C area ratio, no significant differences between mitotic and nonmitotic cells were found using the dish-based analysis for MDA-MB-231 or HCC1806 cells (Fig. S7 in the Supplemental Material). With the pooled analysis of the two cell lines, the redox indices still showed no significant differences in any compartments, but the N:C ratio exhibited a significant increase in mitotic cells when compared to nonmitotic cells [Fig. 7(a)].

Cell-based analysis of MDA-MB-231 or HCC1806 yielded no significant differences in any of the redox indices between nonmitotic and mitotic cells across all compartments (Fig. S8 in the Supplemental Material). However, there was a significant increase in the N:C ratio of mitotic cells compared to nonmitotic cells for both MDA-MB-231 and HCC1806 lines. Pooling HCC1806 and MDA-MB-231 data for cell-based analysis generated consistent results with the dish-based analysis, i.e., no significant changes in redox indices and a significant increase in the N:C ratio of mitotic cells compared to nonmitotic cells (Fig. 7).

Thus, based on both dish and cell-based analyses, the N:C ratio appeared to differentiate between mitotic and nonmitotic cell groups, whereas the ORI indices were not significantly different by large. As indicated in Table S4 in the Supplemental Material, the nuclear area enhancement in mitotic cells contributed more to the N:C ratio increase than the cytoplasmic area, which exhibited little change or increase during mitosis.

**Fig. 8:**
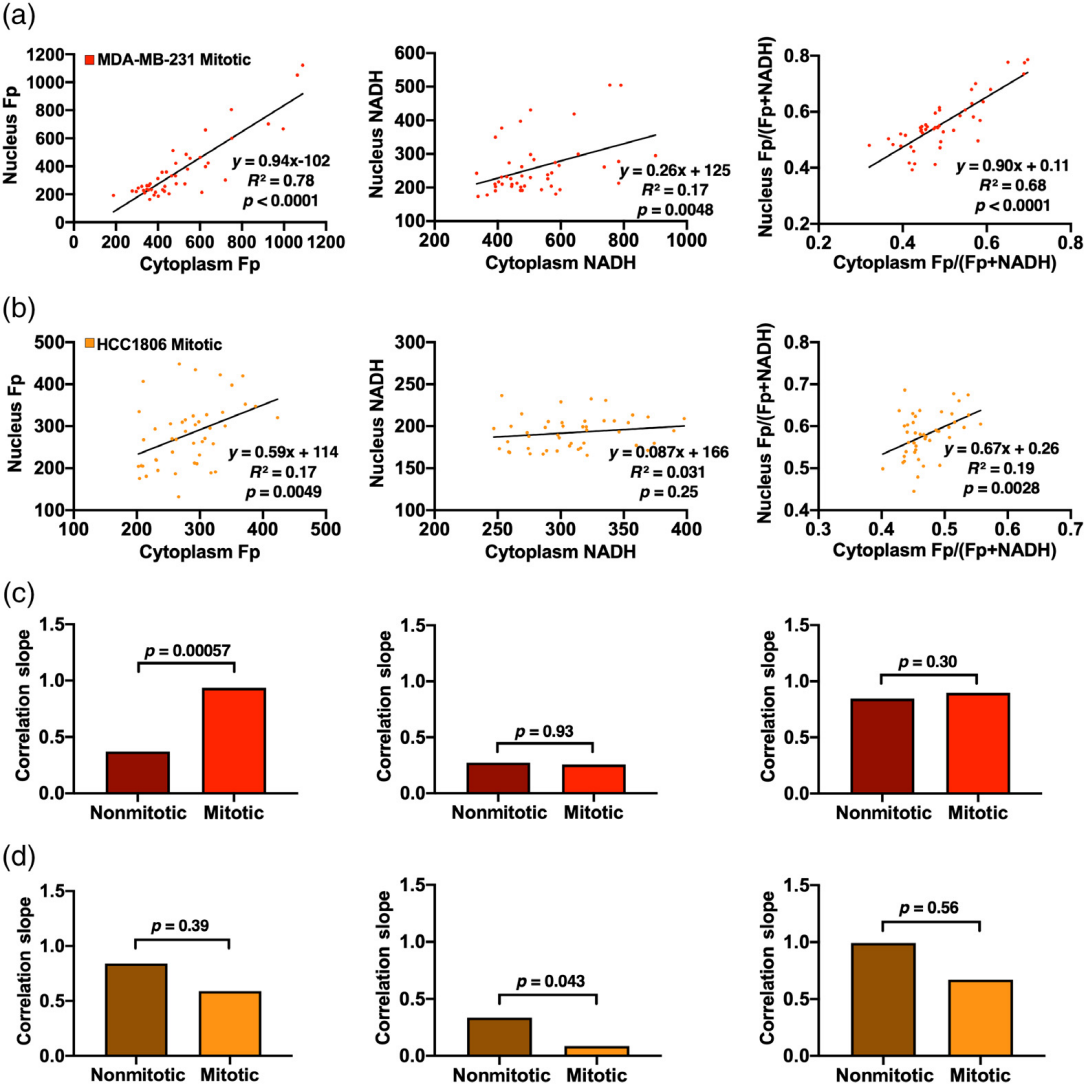
Fp, NADH, and Fp/(Fp + NADH) correlation plots between cytoplasm and nucleus for individual mitotic cells of (a) MDA-MB-231 and (b) HCC1806. Comparison of the correlation slopes between nonmitotic and mitotic cells of (c) MDA-MB-231 and (d) HCC1806. The slopes for nonmitotic and mitotic cells taken from Figs. 3(a) and 3(b) and Figs. 8(a) and 8(b), respectively. First, second, and third columns for Fp, NADH, and Fp/(Fp+NADH), respectively.

We also investigated whether the cytoplasm-nucleus correlation behavior is different between mitotic and nonmitotic cells. The linear correlation plots between nuclear and cytoplasmic redox indices were shown in Figs. 3(a) and 3(b) and Figs. 8(a) and 8(b) for nonmitotic and mitotic TNBC cells, respectively. Comparison of nuclear-cytoplasmic correlation slopes and the statistical significance (p value) for the slope differences between mitotic and nonmitotic cells were presented in Figs. 8(c) and 8(d). The slope for the linear regression between nuclear and cytoplasmic Fp intensities increased from 0.37 in nonmitotic cells to 0.94 in mitotic MDA-MB-231 cells (p<0.001), whereas the nuclear-cytoplasmic correlation slopes for NADH and redox ratio were similar between nonmitotic and mitotic MDA-MB-231 cells (p>0.1). In contrast, HCC1806 mitotic cells have all three nuclear-cytoplasmic correlation slopes decreased compared to nonmitotic HCC1806 cells. However, only the correlation slopes for NADH are significantly different between mitotic and nonmitotic HCC1806 cells (p<0.05) [Fig. 8(d)]. Thus, the cytoplasm-nucleus correlation slopes are different between mitotic and nonmitotic cells in a cell line-dependent manner.

## 4 Discussion

### 4.1 Nuclear Redox Status Compared to Cytoplasmic or Whole-Cell Redox Status

Previous ORI studies utilizing intrinsic fluorescence imaging focus on whole cell or cytoplasmic analysis of fluorescence indices. Quite often, nuclear compartment was removed by thresholding^6^ or lumped together with the cytoplasm^13^^,^^15^ during the data analysis. Few studies investigated the redox status in the nuclear compartment and compared the redox difference between subcellular compartments. In this study, we quantified the nuclear redox status with ORI. Within the tested breast cancer cell lines, we observed that the nuclear compartment has redox indices different from the whole cell or the cytoplasm. Compared to cytoplasm-containing mitochondria, we anticipated lower readings of Fp and NADH in the nucleus. This is corroborated by our observation that the nucleus exhibited a significantly lower or downward trend of Fp and NADH for all four cell lines [Figs. 2(a)–2(d)]. On average, we also found that the nuclear compartment has a more oxidized redox status with a higher redox ratio compared to the cytoplasm in the triple-negative group but not in the receptor-positive group [Figs. 2(c) and 2(f)]. Individual cell-based correlation plots of redox ratios between nuclear and cytoplasm indicate obviously most individual TNBC cells have a higher redox ratio in the nucleus than in the cytoplasm (Fig. 3). Note that redox status depends on cell lines, culture confluency, and other microenvironmental conditions, such as pH and ${\mathrm{pO}}_{2}$, more investigations are needed to test whether the nucleus is more oxidized than cytoplasm in the TNBC cells under other or general experimental conditions.

In addition, there are significant linear correlations of NADH or redox ratios between the nucleus and cytoplasm (Figs. 3 and 4) across and within individual cell lines. This is consistent with that the NADH redox statuses are not isolated or independent between these two subcellular compartments. Interestingly, we found correlations between the nucleus and the cytoplasm Fp when analyzing individual cells within each cell line (Fig. 3), but no correlation was found between the mean nuclear Fp and mean cytoplasmic Fp across four lines [Fig. 4(a)]. While NADH and FAD in the cytoplasm are mainly involved in the mitochondrial metabolism, ${\mathrm{NAD}}^{+}$ and FAD in the nucleus are known to regulate epigenetic and signaling activities that are involved in epithelial–mesenchymal transition in human cancers.^25^^,^^26^ It remains to be investigated whether the nuclear redox status detected by ORI can reflect any underlying biological activities in the nucleus.

### 4.2 Nuclear Redox Status as a Differentiator Between Receptor-Status Groups

The nuclear compartmental redox status has not been investigated for the purpose of cancer biomarker development. With manual segmentation of clearly visible nuclei, we observed a more oxidized nuclear redox status compared to the cytoplasm in the more aggressive triple-negative cells, but nuclear and cytoplasmic redox ratios were similar in the less aggressive receptor-positive cells [Figs. 2(c) and 2(f)]. We also observed that the receptor-positive cell group exhibits significantly higher NADH and significantly lower redox ratio in the nucleus, cytoplasm, and the whole cell compared to the triple-negative group (Fig. 5). This observation is consistent with previous studies demonstrating that triple-negative MDA-MB-231 cells have significantly lower NADH intensity and higher redox ratio than receptor-positive MCF7 and BT474 cells.^6^^,^^13^ This higher redox ratio correlates to the higher invasive potential of breast cancer cells.^13^^–^^15^ Thus, the ORI indices can likely distinguish between breast cancer cells with different aggressiveness based on analysis of not only the whole cell and cytoplasm but also the nucleus. Actually, nuclear ORR differentiated the receptor statuses more significantly than cytoplasmic and whole-cell ORRs. It would be interesting to investigate the biological activities underlying the higher nuclear ORR in the triple-negative cells.

### 4.3 Nuclear to Cytoplasmic Area Ratio Is Not a Good Differentiator Between Breast Cancer Subtypes, But It Is a Better Differentiator of Mitotic and Nonmitotic Cells than ORI Indices

The N:C ratio has been used to differentiate between normal, precancerous, and malignant tissues for both cervical and colon cancers.^19^^,^^21^ In this study, we investigated the utility of the N:C area ratio to differentiate breast cancer subtypes, i.e., receptor statuses, for the first time. We did not observe a significant difference in the N:C ratio between the triple-negative and receptor-positive breast cancer cell groups as well as between cell lines using a dish-based analysis (Fig. 6). There was a significant difference between receptor groups with the use of a cell-based analysis for the N:C ratio, but the effect size was smaller compared to redox indices. Overall, the ORI technique is more useful than the N:C ratio in differentiating between the breast cancer receptor statuses, which are associated with different aggressiveness in breast cancer models and patients.

The cell cycle is an important cellular process in cancer proliferation. Oxidative phosphorylation is induced early in the G1 phase of the cell cycle and remains active during the entire cell cycle.^27^ Previously, a study found an increased NADH/Fp ratio in proliferating cells (undergoing the cell cycle) compared to quiescent cells (under cell cycle arrest).^28^ We have not found any published ORI studies of cell mitosis. We compared mitotic cells (apparently dividing based on nuclear morphology) and nonmitotic cells within the TNBC cell group. We observed that, when using both cell and dish-based analysis for the pooled data from two cell lines, all redox indices did not have significant differences between nonmitotic and mitotic cells in the whole cell, cytoplasm, and nucleus (Fig. 7). Although we found some significant differences in the nuclear-cytoplasmic correlation slopes between mitotic and nonmitotic cells [Figs. 8(c) and 8(d)], the underlying meaning of these correlation slopes remains unknown. Our ORI analysis in this study is based on nonsynchronized cells, which are expected to be distributed in different phases of the cell cycle, and thus may not be sensitive enough to detect changes between mitotic and nonmitotic cells. In the future, we can study the redox status for a specific phase of the cell cycle. Also, fluorescence lifetime imaging microscopy of free and bound NADH in the nucleus may be more suitable to detect subtle changes during the cell cycle.^18^

When pooling data from the two TNBC cell lines, the N:C ratio marked an increase in mitotic cells compared to nonmitotic cells (Fig. 7). These results indicate that the N:C ratio would be a better differentiator between nonmitotic and mitotic cells than the ORI redox indices. The increase in the N:C ratio is consistent with the presence of a larger nuclear area during cell mitosis.

### 4.4 Potential Problems and Limitations

The results of our study rely on the instrument selected and the working hypothesis we have used in this study. In principle, the microscope light power and detector sensitivity may exhibit temporal drift, and calibration with a reference standard is needed during the period of study. However, the latest commercial widefield microscope technology has progressed significantly such that the imaging systems are rather stable in both short and long terms. The solid-state LEDs used in our Zeiss microscope have a lifetime of at least several times longer than metal-halide light bulbs,^29^ and they only turn on for each image acquisition and then turn off after each image acquisition. This is significantly different from classical lamps that stay on continuously in experiments. The LEDs coupled to a Zeiss microscope were found to have perfect short- and long-term stability with the variation (SD) of only 1% to 3.5% during 300 h of illumination.^30^ Also based on our personal communication with the Zeiss technical support and the light source information provided by the Company, Colibri 7 LED adopts an automatic long-term power stabilization mechanism that the “LED power is measured and compared to internal references to allow stable and constant output power over the entire lifetime of each LED.”^29^ Furthermore, with standard procedures of hot pixel management and dark current compensation, the digital Axiocam CMOS camera of the ZEISS microscope is extremely stable in long term without measurable sensitivity drift [Jiangcheng Wang, Carl Zeiss Microscopy, LLC, White Plains, New York, United States, personal communications (2022)]. Therefore, the ZEISS wide-field microscope system has excellent stability and may enable quantitative comparison of biological imaging results during a long-term period without the need for calibration. Consistently, in this study, our direct measurement of the light power and system stability of the Zeiss microscope periodically for 3 months indicated an excellent system stability (Fig. S1 and Tables S1 and S2 in the Supplemental Material), with variations and temporal drifts smaller or even negligible compared to the variability of biological samples (often ∼10% to 20% or more). Nevertheless, to be conservative, we only report a relative percentage of changes >15% as significant results (meanwhile passing the statistical significance test) in this study. Moreover, the comparison of the relative differences of the redox indices between nuclear and cytoplasmic compartments within each cell line should not be affected or be less sensitive to the possible instabilities of light source and detector, and the ORR is also less sensitive to such variations.

We acknowledge that our widefield microscopy approach in this study is limited to two-dimensional (2D) imaging and its spatial resolution may not be ideal for imaging the subcellular details compared to three-dimensional imaging by confocal fluorescence microscopy, which can be utilized to confirm the results of this study in the future. Nevertheless, a widefield microscope has the advantages of accessibility and simplicity with imaging and data analysis and thus can be more cost- and time-effective for certain purposes. Our data in this study demonstrated sufficiently high SNR (>9) for detecting nuclear autofluorescence with higher SNR in the cytoplasm (Table S2 in the Supplemental Material). The statistically significant results demonstrate that 2D imaging acquired through a 20× objective with a widefield fluorescence microscope is sufficient for the purpose to differentiate between subcellular compartments and between breast cancer cell lines. We do not expect our imaging measurements to be overly sensitive to the depth of focus. While the fluorescence intensity and the size of the nucleus may vary with the depth of focus, the cancer cells in 2D cultures are adherent to the glass bottom and relatively flat. The focus adjustment was performed by a single operator. The effects of focus on fluorescence intensity and nuclear size, if there are any, are expected to be consistent and should not significantly affect the relative comparison between cell lines.

In this study, nuclear segmentation was manually performed only on selected cells with clear nuclear boundaries. Despite its shortcomings as an operator-dependent approach subject to artifacts, manual segmentation is a regular analysis approach used in the literature for optical imaging studies of cell cultures.^31^^,^^32^ Because NADH is dominant in mitochondria and mitochondria tend to cluster in the perinuclear region, the nucleus usually exhibits as a clearly visible darker area in the NADH fluorescence images (Fig. 1). Other cells with unclear nuclear morphology were excluded in our analysis. We assumed that the cells with clear nuclear boundaries represent the whole population. Moreover, this study did not segment mitochondrial and cytosolic compartments. Although mitochondrial compartments can be segmented from the cytoplasm by thresholding NADH intensity,^33^ the exact threshold used was based on the judgment of an operator and thus relatively subjective. Under some conditions (e.g., uncoupling or high rate of oxidative phosphorylation), mitochondria can consume more NADH resulting in lower NADH than basal level.^10^^,^^34^ Thus, we did not take the thresholding approach to segment mitochondrial and cytosolic compartments in this study. In the future, it would be interesting to simultaneously image the cellular redox status by ORI and the mitochondrial locations using a fluorescence dye in the third channel. With this approach, it would be possible to investigate the redox statuses among the three subcellular compartments (cytosol, mitochondria, and nucleus) and how they are related to each other and the cancer cell subtypes or aggressiveness.

We also acknowledge that cellular NADPH, expected to be approximately fivefold to tenfold smaller than NADH level,^35^^,^^36^ has the same optical property as NADH, and may contribute to the NADH signal reported in this study. This study is limited to only four breast cancer cell lines. We could investigate more triple-negative and receptor-positive cell lines in the future to check whether the results found in this study hold more generally. The imaging of mitotic cells is also limited to the triple-negative group. With longer incubation time and more cells in the mitotic phase, we can compare the nonmitotic and mitotic cells for the receptor-positive cell lines as well.

## 5 Conclusion

To the best of our knowledge, this is the first study utilizing ORI to characterize and compare the redox status between nuclear and cytoplasmic compartments of cancer cells and compare the ORI indices to the nuclear-cytoplasmic ratio in their potentials as biomarkers for breast cancer aggressiveness. A more oxidized nuclear redox status relative to the cytoplasm is associated with the more aggressive TNBC subtype. There are strong correlations between nuclear and cytoplasmic compartments for the ORI indices for all cell lines, suggesting a redox connection between the subcellular compartments. Optical redox indices of the whole cell, cytoplasm, and nucleus can differentiate between triple-negative and receptor-positive breast cancer groups with different aggressiveness. The N:C ratio does not differentiate between receptor statuses as well as the ORI indices do ; however, it is a better differentiator between nonmitotic and mitotic TNBC cells than the ORI indices. These results indicate the advantage of ORI over the N:C ratio in differentiating breast cancer subtypes with varying aggressiveness.

## Code, Data, and Materials Availability

The MATLAB codes are available upon request.
