# Supplementary material for: Subcellular analysis of nuclear and cytoplasmic redox indices differentiates breast cancer cell subtypes better than nuclear-to-cytoplasmic area ratio
Source: J Biomed Opt. 2022 Aug 9;27(8):086001. doi: 10.1117/1.JBO.27.8.086001 (PMC9360498; doi:10.1117/1.JBO.27.8.086001)
Supplement: Supplementary file 1 [file JBO_027_086001_SD001.pdf]

Supplementary material for Jacob *et al.* "Subcellular analysis of nuclear and cytoplasmic redox indices differentiates breast cancer cell subtypes better than nuclear to cytoplasmic area ratio"

Methods

Power measurement of LED light sources: The illumination power of LED light sources was measured with the 10×, 0.3 NA PH objective using a Thorlabs meter (PM 100A) and sensor (S170C) during a period of 3 months. Room lights were off for all measurements. This sensor is embedded in a metal frame that has the dimensions of a standard microscope slide (1 × 3 inches), so it can be mounted on the stage like an ordinary slide. Procedure for positioning the sensor is based on that developed by the QUAREP-LiMi working group 1. See https://www.protocols.io/view/illumination-power-and-illumination-stability-5jyl853ndl2w/v1. Briefly, a standard Leica convallaria slide was used as a sample to position the focus position of the objective lens. After focusing on the specimen using transmitted light illumination, the condenser lens was positioned for Kohler illumination. The field diaphragm was opened so that the illuminated area was a circle with diameter ~ 5 mm. The convallaria slide was removed and replaced with the sensor, with the sensitive area facing down. A target etched on the back of the metal frame indicates the position of the light-sensitive area on the front of the frame; this target was positioned so that the circular transmitted light illumination was centered on it. The transmitted light was turned off during all power measurements. The meter was set to the desired wavelength and zeroed once, at the beginning of each measurement session. The system was found to be very stable in the near term and the power reading did not change within each session. So only one reading was obtained from each measurement session. Measurements were done for four light wavelengths (385, 475, 555, 630 nm) at various intensity levels (5%, 10%, 25%, 50%, 65%, 80%, 100%). In total eight measurement sessions covered a period of three months. The measurements for a specific wavelength and an intensity level were averaged across different time points to obtain mean ± SD. The coefficients of variations (CV) were calculated by SD/mean. The temporal drifts within 100 days were calculated based on the slopes from the linear regression analysis of the power measurements versus days using Prism 9. The p values for the significance of non-zero slopes were also obtained.

Stability test of Zeiss microscope with standard samples: Stock solutions of 100 µM NADH or FAD dissolved in PBS (pH7.0 with Mg^{2+} and Ca^{2+}) were made and aliquoted into a few 1.5 ml bullet tubes wrapped by aluminum foil and stored in -80 ^{0}C freezer. Frozen NADH and FAD solutions were warmed up to dissolve before each stability imaging session. 500 µl NADH solution, FAD solution, and blank PBS buffer (pH 7, stored under room temperature) were separately added to a chamber in a 35mm four-chamber glass-bottom dish (Cellvis, Sunnyvale, CA, USA. Product #: D35C4-20-1.5-N). Efforts were made to reduce the exposure of NADH and FAD solutions to surrounding light by aluminum foil wrapping and a black box container during preparation and transportation to the ZEISS microscope facility. In total nine imaging sessions were performed during a period of 3 months. For each imaging session, two to three dishes were imaged under 37 ^{0}C using the same instrument settings for cell cultures. Three FOVs were imaged per chamber. Focus was consistently adjusted based on an image of a small Greek letter inscribed by a black permanent marker in the central area of the glass bottom inside

surface. The image raw data were processed with a home-made Matlab program to obtain the mean fluorescence in ROIs that were manually drawn by the operator. Occasionally FOVs with extremely abnormal results were discarded as outliers or due to technical issues. The average values of DAPI and EGFP channel signals at each imaging session were obtained by averaging across all FOVs (n=5-9). These signals were then averaged across the nine imaging sessions to obtain the overall mean and standard deviations (SD). The coefficients of variations (CV) were calculated by SD/mean. The temporal signal drifts within 100 days were calculated based on the slopes from the linear regression analysis of the fluorescence versus days using Prism 9. The p values for the significance of non-zero slopes were obtained.

Figures

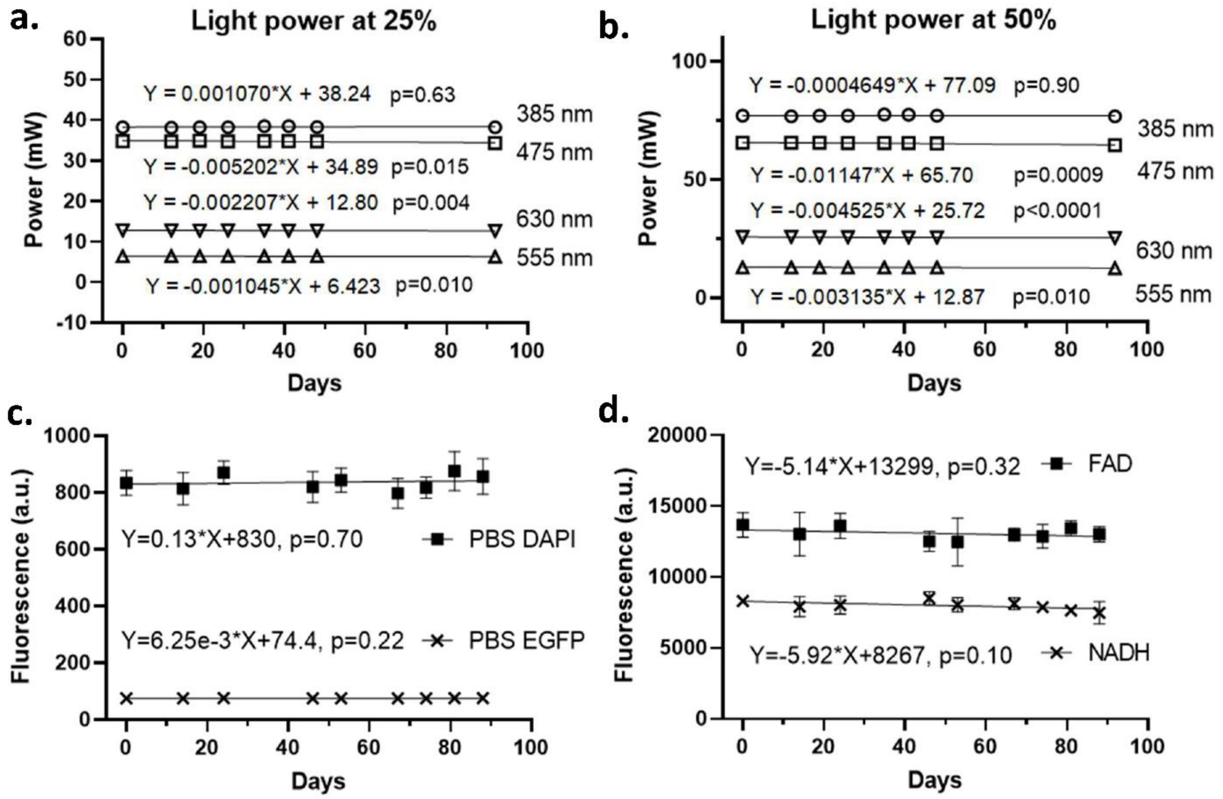

Figure S1. Stability test of ZEISS Axio Observer 7 wide-field microscope. a-b) Power of LED light sources at different wavelengths (385, 475, 555, 630 nm) illuminated at 25% (a) and 50% (b); c) The DAPI and EGFP channel fluorescence of PBS buffer; d) Fluorescence of 100 uM NADH and FAD solutions. Linear regression equations and the p values of the non-zero slopes displayed beside corresponding data lines. Mean ± SD (N=5-9 FOVs).

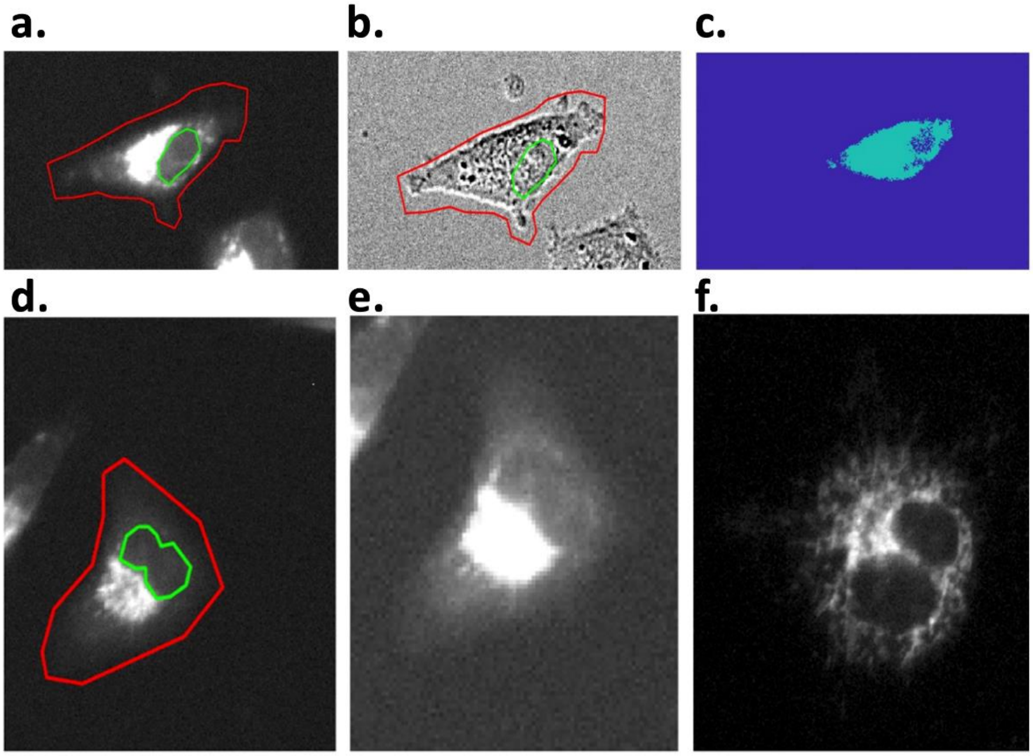

Figure S2 Typical cell images for analysis. (**a-c**) Depiction of ROIs drawn for both whole cell (red) and nucleus (green) on a representative MDA-MB-231 cell. (a) NADH raw image used for drawing ROI, (b) corresponding white light image, and (c) whole cell mask generated by thresholding at SNR≥7.5 without contour. (**d-f**) Mitotic cell images of MDA-MB-231 and HCC1806. (d) Depiction of ROIs drawn for both whole cell (red) and nucleus (green) on an NADH image of a mitotic MDA-MB-231 cell. (e) Zoomed-in NADH image of the MDA-MB-231 cell, contour not shown. (f) NADH image of a mitotic HCC1806 cell indicating division of nuclei, mostly likely in the stage of telophase.

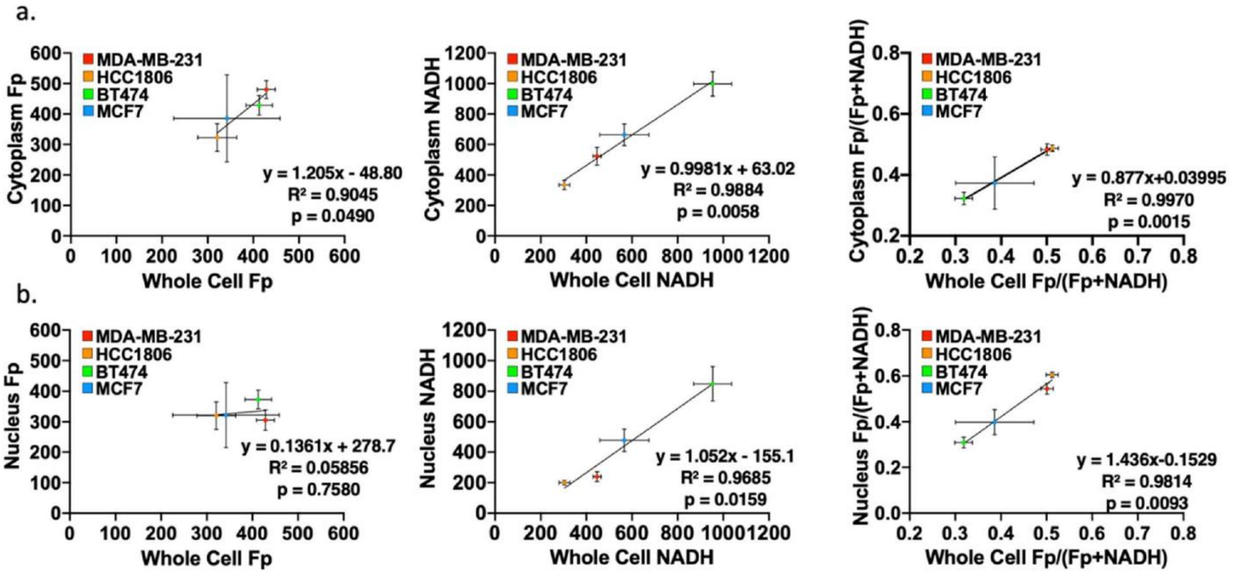

Figure S3 Correlation plots of Fp, NADH, and Fp/(Fp+NADH) between subcellular regions and whole cell across 4 breast cancer cell lines. (a) Cytoplasm vs whole cell, and (b) Nucleus vs whole cell.

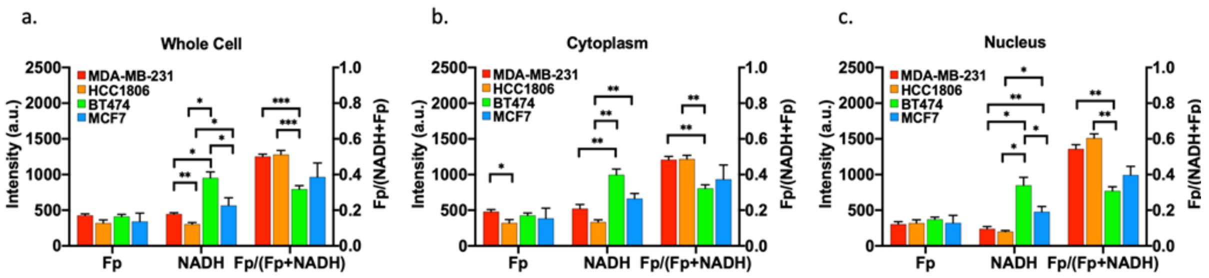

Figure S4 Comparison between cell lines of redox indices using dish-based analysis (N=3, mean ± SD). (a) Whole cell, (b) Cytoplasm, and (c) Nucleus, ***p < 0.001, **p < 0.01, *p < 0.05.

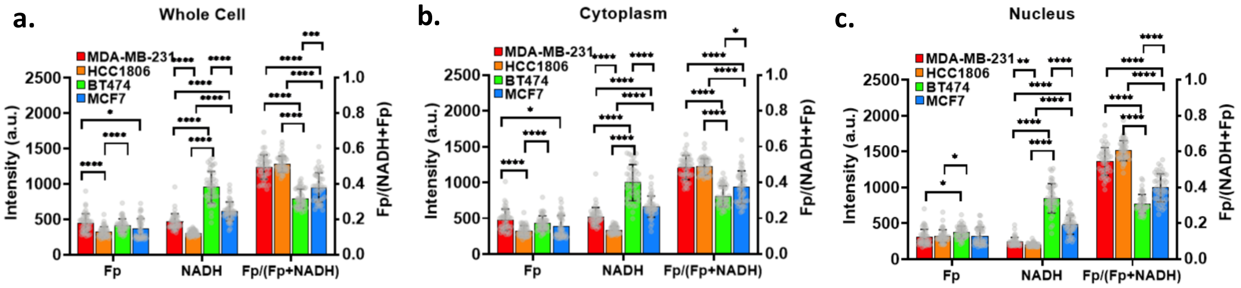

Figure S5 Comparison between cell lines of redox indices using cell-based analysis (N=45, mean ± SD). (a) Whole cell, (b) Cytoplasm, and (c) Nucleus, ****p < 0.0001, **p < 0.01, *p < 0.05. Only the percentage differences in mean values greater than 15% were marked with statistical significance.

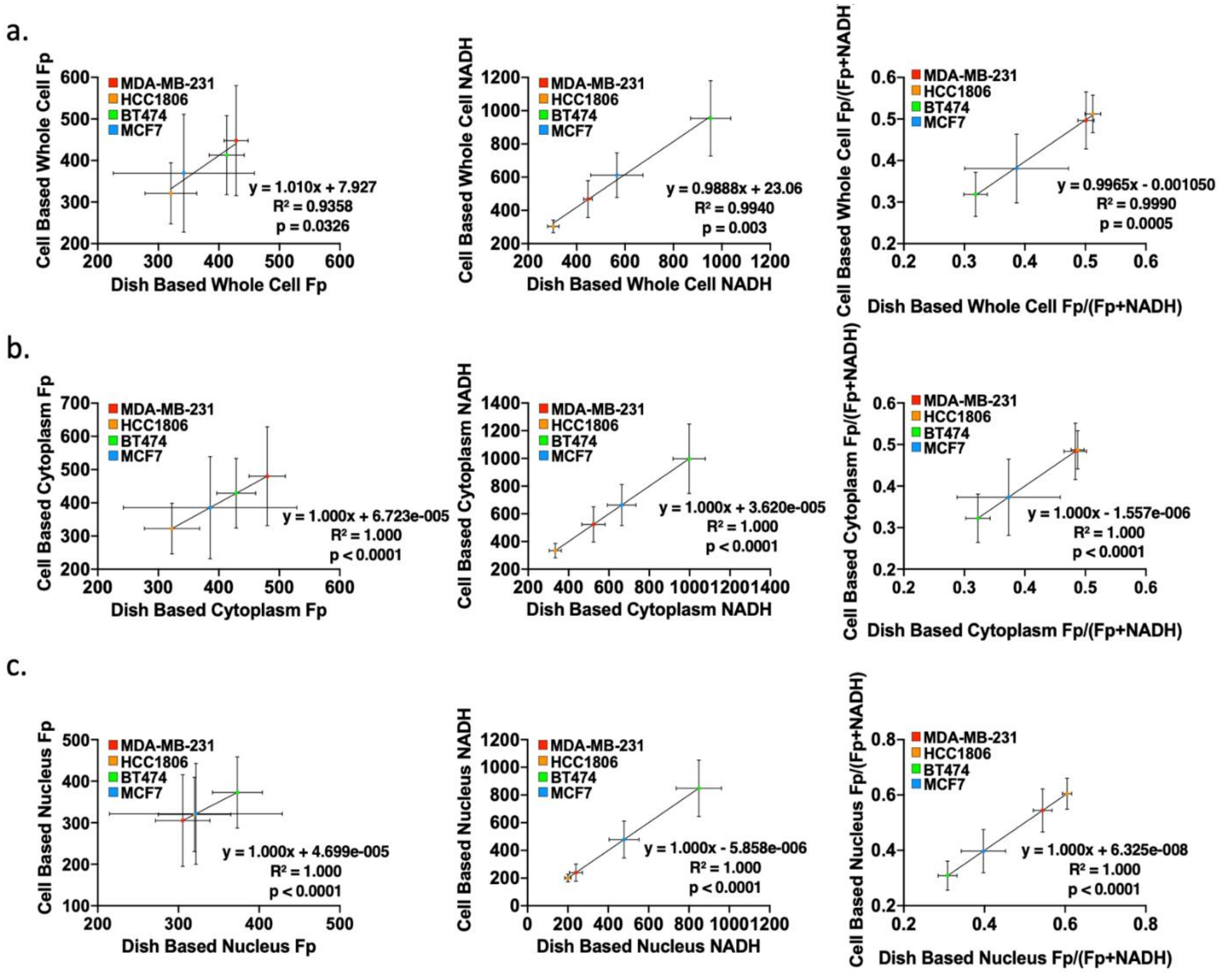

Figure S6 Correlation plots of dish-based vs cell-based redox indices for MDA-MB-231, HCC1806, BT474, MCF7. (a) Whole cell redox indices, (b) Cytoplasm redox indices, and (c) Nucleus redox indices. mean ± SD.

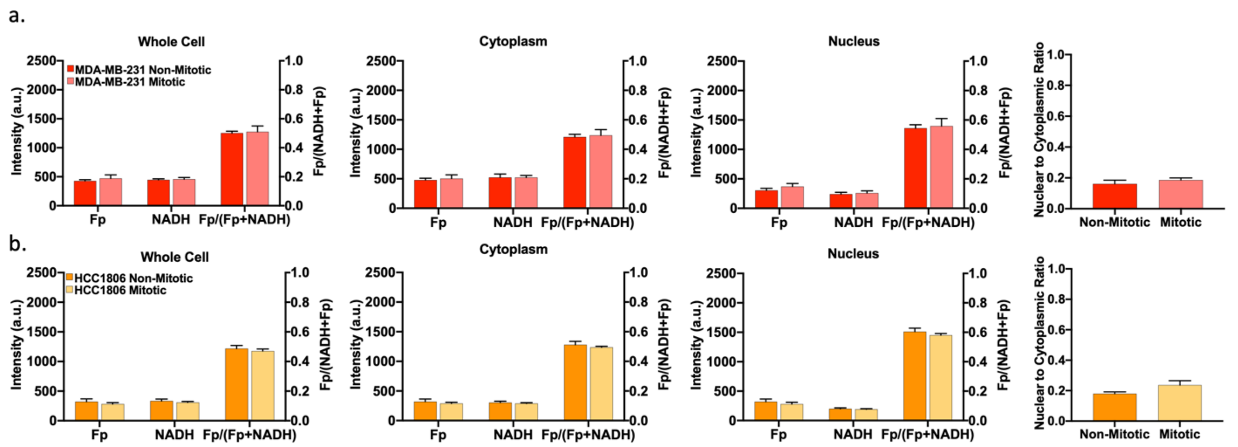

Figure S7 Comparison of non-mitotic and mitotic cells using dish-based analysis (N=3, mean ± SD). (a) MDA-MB-231 non-mitotic compared to mitotic cells for whole cell, cytoplasm, and nucleus redox indices and nuclear to cytoplasmic area ratio. (b) HCC1806 non-mitotic compared to mitotic cells for whole cell, cytoplasm, and nucleus redox indices and nuclear to cytoplasmic area ratio.

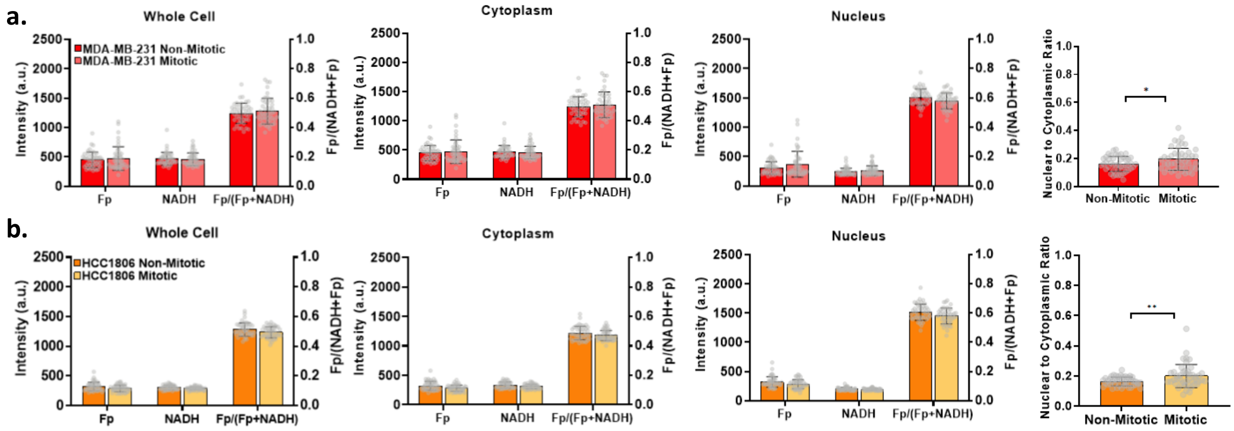

Figure S8 Comparison of non-mitotic and mitotic cells using cell-based analysis (N=45, mean ± SD). (a) MDA-MB-231 non-mitotic compared to mitotic cells for whole cell, cytoplasm, and nucleus redox indices and nuclear to cytoplasmic ratio. (b) HCC1806 non-mitotic compared to mitotic cells for whole cell, cytoplasm, and nucleus redox indices and nuclear to cytoplasmic ratio. For HCC1806, the lower Fp and NADH across all compartments in mitotic cells compared non-mitotic cells were statistically significant, but the differences did not exceed the reliability threshold of 15% set in this study.

Tables

Table S1 Zeiss microscope light power stability during a period of 3 months.

Light Intensity	Coefficients of Variation (%)				Temporal drift 100 days (%)			
	**385 nm**	**475 nm**	**555 nm**	**630 nm**	**385 nm**	**475 nm**	**555 nm**	**630 nm**
**5%**	0.45	0.79	0.76	0.54	0.32	-1.43	-2.23	-1.60
**10%**	0.43	0.64	0.82	0.21	0.42	-1.41	-2.40	-0.61
**25%**	0.39	0.52	0.55	0.56	0.28	-1.49	-1.63	-1.72
**50%**	0.33	0.53	0.83	0.51	-0.06	-1.75	-2.44	-1.76
**65%**	0.35	0.54	0.70	0.49	-0.04	-1.84	-2.04	-1.66
**80%**	0.35	0.57	0.81	0.47	0.04	-1.96	-2.66	-1.60
**100%**	0.34	0.57	0.66	0.45	0.02	-1.93	-2.08	-1.55

Table S2 Stability test of the Zeiss microscope using PBS buffers and 100 µM NADH and FAD solutions (mean ± SD, N=5-9).

Parameters	PBS DAPI	PBS EGFP	NADH	FAD
**Mean**	836	75	7973	13043
**SD**	26	0.40	298	407
**CV (%)**	3.06	0.54	3.74	3.12
**Temporal drift in 100 days (%)***	1.57	0.84	-7.16	-3.87

*All slopes of drifts were not significantly different from zero ($p > 0.05$, Figure S1)

Table S3 Signal to Noise Ratios (SNR ± SD) of FAD and NADH for the whole cell, cytoplasm, and nucleus in each cell line. SNRs were averaged over five cells in each FOV, and then averaged over the three field of views (FOV) in each dish and then were averaged over three dishes (N=3).

Cell Lines	ORI Channels	Whole Cell	Cytoplasm	Nucleus
**MDA-MB-231**	**FAD SNR**	27 ± 2.5	29 ± 2.5	18 ± 2.4
	**NADH SNR**	24 ± 2.6	27 ± 2.7	13 ± 1.6
**HCC1806**	**FAD SNR**	16 ± 2.1	16 ± 3.2	16 ± 2.8
	**NADH SNR**	16 ± 0.7	16 ± 3.2	9.5 ± 1.6
**BT474**	**FAD SNR**	25 ± 2.4	26 ± 2.6	22 ± 2.5
	**NADH SNR**	50 ± 3.9	52 ± 3.5	44 ± 5.7
**MCF7**	**FAD SNR**	23 ± 0.7	13 ± 2.3	11 ± 2.4
	**NADH SNR**	32 ± 1.3	32 ± 5.0	23 ± 1.6

Table S4 Nuclear and cytoplasmic areas ± SD for non-mitotic versus mitotic cell lines. Areas were averaged over five cells in each FOV, and then averaged over the three field of views (FOV) in each dish and then were averaged over three dishes (N=3).

Cell Lines		Nuclear Area (pixels)	Cytoplasmic Area (pixels)	Nuclear to Cytoplasmic Ratio
**MDA-MB-231**	**Non-mitotic**	934 ± 4	6284 ± 626	0.16 ± 0.02
	**Mitotic**	1347 ± 104	8714 ± 788	0.182 ± 0.02
	**Change (%)**	44.2	38.7	13.0
	**p**	0.020	0.015	0.33
**HCC1806**	**Non-mitotic**	2142 ± 170	12124 ± 1816	0.182 ± 0.02
	**Mitotic**	2331 ± 252	10907 ± 698	0.24 ± 0.03
	**Change (%)**	8.5	-1.6	31.1
	**p**	0.36	0.94	0.061
